# A federated learning differential privacy algorithm for non-Gaussian heterogeneous data

**DOI:** 10.1038/s41598-023-33044-y

**Published:** 2023-04-10

**Authors:** Xinyu Yang, Weisan Wu

**Affiliations:** School of Mathematics and Statistics, Baicheng Normal University, Baicheng, 137000 China

**Keywords:** Computational science, Computer science, Information technology, Statistics

## Abstract

Multi-center heterogeneous data are a hot topic in federated learning. The data of clients and centers do not follow a normal distribution, posing significant challenges to learning. Based on the assumption that the client data have a multivariate skewed normal distribution, we improve the **DP-Fed-mv-PPCA** model. We use a Bayesian framework to construct prior distributions of local parameters and use expectation maximization and pseudo-Newton algorithms to obtain robust parameter estimates. Then, the clipping algorithm and differential privacy algorithm are used to solve the problem in which the model parameters do not have a display solution and achieve privacy guarantee. Furthermore, we verified the effectiveness of our model using synthetic and actual data from the Internet of vehicles.

## Introduction

In the present increasingly developed technology, various intelligent terminals collect personal data, and because of improvements in storage devices and communication technology, people have begun to focus on the collection and release of high-dimensional complex data; however, the data often contain private information. Considering this situation, Google proposed the federated learning (FL) model^1^ such that the clients do not send the original data to the central server, but only need to train the data locally and thereafter send the trained parameters to the central server. Although the raw data are not sent, an attacker can still infer the original data based on the sent training parameters, thereby causing privacy leakage. To ensure client-level privacy, the fashion method uses differential privacy (DP) with rigorous mathematical proofs. Several successful algorithms have combined DP and FL.

Heterogeneous and heavy-tailed data are often encountered in high-dimensional data, and the effects of traditional privacy protection algorithms on such data are often unsatisfactory. To protect client privacy, researchers have attempted to combine DP with different FL models such as those of Agarwal et al.^2^, Asoodeh et al.^3^, and Geyer et al.^4^. In 2022, Balelli adopted a DP-FL algorithm (**DP-Fed-mv-PPCA**)^5^ for multi-view data. The algorithm assumes that data and latent variables follow a Gaussian distribution, handles statistical heterogeneity and missing view data in local datasets using the Bayesian method, and proves that it guarantees privacy and convergence. FL can be used in various fields, such as using FL in combination with the Internet of Things, edge computing blockchains, and 5G networks, as well as for studying more complex instances with heterogeneous servers.^6–8^. Simultaneously, we also noted that in the field of vehicle networks, the use of FL methods can not only effectively integrate data generated by various users and vehicles but also protect users’ personal privacy. There have been some detailed studies on this subject^9–11^, but they are all based on the strong assumption that the data follow a normal distribution, whereas in practice, the data are often asymmetric, heavy-tailed, or long-tailed.

In this study, we use a multivariate skewed normal distribution instead of a Gaussian distribution to improve the robustness of the statistical inference model for client data with heavy-tail and asymmetric characteristics, and to ensure the privacy of client data. A skewed normal distribution was proposed by Azzalini^12^ in 1985 on the basis that the data did not meet the requirements of the normal distribution. This distribution has a flexible skewness parameter that describes data asymmetry. After the bias normal distribution was proposed, remarkable success was achieved in data fitting. Based on this, Azzalini proposed a multivariate normal distribution^13^ in 1996 and skewed distribution^14^ in 2005. Subsequently, new skewed distributions have been proposed continuously, for instance, skew-laplace distribution^15^, skew-cauchy distribution^16^, and skew-logistic distribution^17^. The main purpose of these distributions is to describe the degree of skew and thicker tail of the data by relying on flexible hyperparameters.

The remainder of this paper is organized as follows: In “Related works” section, we present the work on heterogeneous FL and the contributions of our work. In “Preliminaries” section, we introduce some necessary concepts, theories, and notation symbols, and provide a detailed analysis of client data with skewed normal distribution, combined with the design based on these conclusions. In “Methods” section, we skew the normal distribution difference of the privacy federal study expectation maximization (EM) algorithm. Furthermore, we verify the effect of our model using synthetic and real data from the Internet of Vehicles in “Performance evaluation” section. Finally, “Conclusions” section summarizes the advantages of the model and discusses future research.

## Related works

Several studies have been conducted on heterogeneous data federation learning. The FedProx algorithm proposed by Li et al. in 2018^18^ is an improved FedAvg algorithm for partial local work that avoids data heterogeneity by introducing an approximation term. Li considered multi-center scenarios in 2020^19^. In the past two years, Zhu considered the knowledge distillation method in 2021^20^ and Guo studied heterogeneous FL in 5G networks using the dynamic scheduling method in 2022^21^. Similarly, Wang also developed the device sampling method of heterogeneous FL and the node sampling theory of graph convolutional neural network in 2021^22^. Shen studied a fast algorithm for heterogeneous federation learning in 2022^23^ when clients were mixed, which could effectively reduce the variance of the model.

For more theoretical and applied research, please read Fed2KD: Heterogeneous FL for Pandemic Risk Assessment via Two-way Knowledge Distillation by Sun et al.^24^,HFedMS: Heterogeneous FL with Memorable Data Semantics in Industrial Metaverse by Zeng et al.^25^, FedRolex: Model-heterogeneous FL with Rolling Sub-model Extraction by Alam et al.^26^, Lazy Aggregation for Heterogeneous FL by Xu et al.^27^ and FedFOR: Stateless Heterogeneous FL with First-order Regularization by Tian et al.^28^ in detail.

All these studies are generalized studies on heterogeneous FL, either to improve computing speed or to focus on the heterogeneity of the client or central server. However, there have been no in-depth studies on irregular data with non-normal scores. Therefore, our study contributes to the literature in several ways.

- We use the EM algorithm to study the parameter estimation problem of client and server data with biased normal structure FL.
- By calculating the sensitivity of the parameters, our algorithm makes the parameters have a small estimation error and ensures the differential privacy during the EM algorithm iteration.
- We applied our algorithm to both synthetic data and real data from the Internet of vehicles. The results demonstrate that our algorithm is more robust and can ensure the accuracy of estimation while achieving DP.

## Preliminaries

### Model setting

We assume that there are *I* mutually independent service centers and that each service center $i, i\in \{1,2,\cdots ,I\}$ contains the local original dataset $D_i =\{x_{i,n}\}_{i=1}^{n_i}$. Furthermore, we assume that the local dataset is incomplete and missing at least one view in the dataset. For every $g\in {1,\cdots ,G}$, the dimensions of the $gth$view are $d_g$, and $d :=\sum _{g=1}^G d_g$. We denote by $x_{i,n}^{(g)}$ the raw data of subject *n* in center *i* corresponding to the $gth$-view, and $x_{i,n} =(x_{i,n}^{(1)},\cdots ,x_{i,n}^{(g)})$.

### Federated multi-views PPCA and multivariate skew normal distribution

In this section, we first review the federated multi-view PPCA model given by Balelli^5^ and assume that the client data have a multivariate skewed normal distribution. Then, we present related concepts and lemmas. Based on Arella noValle’s study^29^, we extend the theories of joint density and conditional density of multivariate skew normal random variables.

We consider a situation in which the underlying data distribution has a hierarchical structure and assume that the global parameter is ${\tilde{\theta }}$ and the corresponding density function is $f({\tilde{\theta }})$. For each center, the local parameter $\theta _i$ is generated by the distribution $f(\theta _i|{\tilde{\theta }})$. Furthermore, the local data $x_i$ are generated from the local distribution $f(x_i|\theta _i)$. The federated algorithm, known as **Fed-mv-PPCA**, can be used to solve the inverse problem from the local data to the central server in a hierarchical structure using a Bayesian method, and the parameters of this model can be estimated by maximizing the likelihood.

First, we assume that the local data of subject *n* corresponding to the $gth$view, $x_{i,n}^{(g)}$, are generated as follows:

1 $$\begin{aligned} x_{i,n}^{(g)} = \mu _i^{(g)} + \varepsilon _i^{(g)} +A_i^{(g)}z_{i,n}, \end{aligned}$$

where $\varepsilon _i^{(g)} \sim N(0,{\sigma _i^{(g)}}^2 I_{d_g})$ denotes a Gaussian noise, $z_{i,n} \sim SN_{d_g}(0,I,\Lambda _i^{(g)})$ denotes a $q-$dimension latent variable, and we assumed $q< \min _g {d_g}$ for computation. $A_i^{(g)}$ and $\mu _i^{(g)}$ are the coefficients and offsets with respect to view *g*, respectively. We find that $A_i^{(g)}z_{i,n}$ follows the multivariate skew normal distribution by Arellano Valle (2005) in Corollary 2.3 as follows^29^:

2 $$\begin{aligned} A_i^{(g)}z_{i,n}\sim SN_{d_g}(0,A_i^{(g)}{A_i^{(g)}}^T\Lambda _i^{(g)}), \end{aligned}$$

and

3 $$\begin{aligned}{} & {} \varepsilon _i^{(g)} + \mu _i^{(g)} \sim N_{d_g}(\mu _i^{(g)},{\sigma _i^{(g)}}^2 I_{d_g}), \end{aligned}$$

4 $$\begin{aligned}{} & {} x_{i,n}^{(g)} \sim SN_{d_g}(\mu _i^{(g)},\Sigma _i^{(g)},{\tilde{\Lambda }}_i^{(g)}), \end{aligned}$$

where ${\tilde{\Lambda }}_i^{(g)} = (1+\eta ^T\Omega _X^{-1}\eta )^{-\frac{1}{2}}\omega _X \Omega _X^{-1}\Omega \eta , \eta =\omega ^{-1}A_i^{(g)}\Lambda _i^{(g)},\omega _X=(\Omega _X\odot I)^{\frac{1}{2}}, \Sigma _i^{(g)}= A_i^{(g)}{A_i^{(g)}}^T +{\sigma _i^{(g)}}^2 I_{d_g} ,$ and $\odot$ is the Hadamard product. Then, concatenate all views $x_{i,n}^{(g)}$ into a compact formulation from Equation (1) as follows:

5 $$\begin{aligned} x_{i,n} = \mu _i + \Phi _i +A_iz_{i,n}, \end{aligned}$$

where $A_i,\mu _i$ are obtained by concatenating all $A_i^{(g)},\mu _i^{(g)}$, and $\Phi _i$ denotes a block diagonal matrix with the $gth$block from $\varepsilon _i^{(g)}.$ From the described analysis process, we can denote the local parameter as follows:

6 $$\begin{aligned} \theta _i:=\{\mu _i^{(g)},A_i^{(g)},{\sigma _i^{(g)}}^2, \Lambda _i^{(g)}\}. \end{aligned}$$

We consider a Bayesian approach to (5) in which $\theta _i$ is regarded as random with a prior distribution that reflects the degree of belief in the different values of these quantities. The prior distributions selected are weakly informative and subject to vague prior knowledge, and this avoids non-integrable posterior distributions. The prior distributions for the model are as follows:

7 $$\begin{aligned} \begin{aligned} (\mu _i^{(g)}|{\tilde{\mu }}^{(g)},\sigma _{{\tilde{\mu }}}) \sim&N_{d_g}({\tilde{\mu }}^{(g)},{\sigma _{{\tilde{\mu }}}}^2),\\ (A_i^{(g)}|{\tilde{A}}^{(g)},\sigma _{{\tilde{A}}^{(g)}}) \sim&N_{d_g}({\tilde{A}}^{(g)},{\sigma _{{\tilde{A}}^{(g)}}}^2),\\ ({\sigma _i^{(g)}}^{-2}|{\tilde{\beta }}^{(g)},{\tilde{\gamma }}^{(g)}) \sim&\Gamma ({\tilde{\beta }}^{(g)},{\tilde{\gamma }}^{(g)}),\\ (\delta (\lambda _i^{(g)})| \lambda ^{(g)}) \sim&U(-1,1). \end{aligned} \end{aligned}$$

Thus, we denote the global prior distribution by parameter ${\tilde{\theta }}_i = \{{\tilde{\mu }}^{(g)},\sigma _{{\tilde{\mu }}},{\tilde{A}}^{(g)},\sigma _{{\tilde{A}}^{(g)}}, {\tilde{\beta }}^{(g)},{\tilde{\gamma }}^{(g)},{\tilde{\Lambda }}^{(g)} \}$.

Second, we need to concrete analyze the joint and conditional density functions of the observed and latent variables. Therefore, we introduce the Arellano Valle 2005 standard skewed normal distribution theory and extend it to general location and scale cases^30^.

#### Lemma 3.1

If $Z_1\sim SN_m(\Lambda _1),Z_2\sim SN_m(\Lambda _2)$, then the conditional density of $Z_1$ given $Z_2=z_2$ is as follows:

8 $$\begin{aligned} f_{Z_1|Z_2=z_2}(z_1) = \phi _{m_1}(z_1)\Phi _{m_1+m_2}(\Lambda _1^Tz_1+\Lambda _2^Tz_2|I_{m_1+m_2}-\Lambda _1^T\Lambda _1 - \Lambda _2^T\Lambda _2)/\Phi _{m_2}(\Lambda _2^Tz_2|I_{m_2}-\Lambda _2^T\Lambda _2 ) \end{aligned}$$.

#### Theorem 3.2

If $W_1 = \mu _1 +\Sigma _1^{\frac{1}{2}}Z_1,Z_1\sim SN_{m1}(\Lambda _1), W_2 = \mu _2 +\Sigma _2^{\frac{1}{2}}Z_2,Z_1\sim SN_{m2}(\Lambda _2)$, then the conditional density of $W_1$ given $W_2=w_2$ is as follows:

9 $$\begin{aligned} \begin{aligned} f_{W_1|W_2=w_2}(w_1) =&|\Sigma _1-\Sigma _{12}\Sigma _2^{-1}\Sigma _{12}|^{-\frac{1}{2}}|\Sigma _2|^{-\frac{1}{2}} \phi _{m_1}(\Sigma _1^{-\frac{1}{2}}(w_1-\mu _1))\\&\Phi _{m_1+m_2}(\Lambda _1^T\Sigma _1^{-\frac{1}{2}}(w_1-\mu _1)+\Lambda _2^T\Sigma _2^{-\frac{1}{2}}(w_2-\mu _2)|I_{m_1+m_2}-\Lambda _1^T\Lambda _1 - \Lambda _2^T\Lambda _2)/\Phi _{m_2}(\Lambda _2^T\Sigma _2^{-\frac{1}{2}}(w_2-\mu _2)|I_{m_2}-\Lambda _2^T\Lambda _2 ). \end{aligned} \end{aligned}$$

#### Proof

Because of $W_1 = \mu _1 +\Sigma _1^{\frac{1}{2}}Z_1,Z_1\sim SN_{m1}(\Lambda _1), W_2 = \mu _2 +\Sigma _2^{\frac{1}{2}}Z_2,Z_1\sim SN_{m2}(\Lambda _2)$, we know about $W_1 \sim SN_{m1}(\mu _1, \Sigma _1,\Lambda _1)$, $W_2 \sim SN_{m2}(\mu _2, \Sigma _2,\Lambda _2)$. Thus, the random variable $W_1$ has the following density function:

10 $$\begin{aligned} \begin{aligned} f_{W_1^*}(w_1) =&2^m |\Sigma _1|^{-\frac{1}{2}}\phi _{k_1}(\Sigma _1^{-\frac{1}{2}}(w_1-\mu _1))\Phi _{m_1}(\Lambda _1^T\Sigma _1^{-\frac{1}{2}}(w_1-\mu _1)|I_{m_1}-\Lambda _1^T\Lambda _1 )f_{W_2^*}(w_2)\\ =&2^m |\Sigma _2|^{-\frac{1}{2}}\phi _{k_2}(\Sigma _2^{-\frac{1}{2}}(w_2-\mu _2))\Phi _{m_2}(\Lambda _2^T\Sigma _2^{-\frac{1}{2}}(w_2-\mu _2)|I_{m_2}-\Lambda _2^T\Lambda _2 ). \end{aligned} \end{aligned}$$

$W_1,W_2$ have a joint density function, as follows:

11 $$\begin{aligned} \begin{aligned} f_{W_1,W_2}(w_1,w_2) =&2^{m_1+m_2}|\Sigma _1-\Sigma _{12}\Sigma _2^{-1}\Sigma _{12}|^{-\frac{1}{2}}|\Sigma _2|^{-\frac{1}{2}}\phi _{m_1}(\Sigma _1^{-\frac{1}{2}}(w_1-\mu _1))\phi _{k_2}(\Sigma _2^{-\frac{1}{2}}(w_2-\mu _2))\\&\Phi _{m_1+m_2}(\Lambda _1^T\Sigma _1^{-\frac{1}{2}}(w_1-\mu _1)+\Lambda _2^T\Sigma _2^{-\frac{1}{2}}(w_2-\mu _2)|I_{m_1+m_2}-\Lambda _1^T\Lambda _1 - \Lambda _2^T\Lambda _2). \end{aligned} \end{aligned}$$

Furthermore, the conditional density function of $W_1$ given $W_2=w_2$ is as follows:

12 $$\begin{aligned} \begin{aligned} f_{W_1|W_2=w_2}(w_1) = \frac{\phi _{m_1}(\Sigma _1^{-\frac{1}{2}}(w_1-\mu _1))\Phi _{m_1+m_2}(\Lambda _1^T\Sigma _1^{-\frac{1}{2}}(w_1-\mu _1)+\Lambda _2^T\Sigma _2^{-\frac{1}{2}}(w_2-\mu _2)|I_{m_1+m_2}-\Lambda _1^T\Lambda _1 - \Lambda _2^T\Lambda _2)}{|\Sigma _1-\Sigma _{12}\Sigma _2^{-1}\Sigma _{12}|^{\frac{1}{2}}|\Sigma _2|^{\frac{1}{2}}\Phi _{m_2}(\Lambda _2^T\Sigma _2^{-\frac{1}{2}}(w_2-\mu _2)|I_{m_2}-\Lambda _2^T\Lambda _2 )}. \end{aligned} \end{aligned}$$

Specifically, we can express the $(x_{i,n}, z_{i,n})$ joint density as follows:

13 $$\begin{aligned} \begin{aligned} f(x_{i,n}, z_{i,n})=&2^{d+q}|A_iA_i^T +\sigma _i^2 I_{d_g}|^{-\frac{1}{2}} \phi _{d_g}(|A_iA_i^T +\sigma _i^2 I_{d_g}|^{-\frac{1}{2}}(x_{i,n}-\mu _i))\phi _q(z_{i,n})\\&\Phi _{d_g+q}({\tilde{\Lambda }}_i^T |A_iA_i^T +\sigma _i^2 I_{d_g}|^{-\frac{1}{2}}(x_{i,n}-\mu _i) +{\tilde{\Lambda }}_i^T z_{i,n}|I_{d_g+q}-{\tilde{\Lambda }}_i^T{\tilde{\Lambda }}_i - \Lambda _i^T\Lambda _i)\\ =&2^{d+q}|A_iA_i^T +\sigma _i^2 I_{d_g}|^{-\frac{1}{2}} 2^d \exp {-\frac{1}{2}(x_{i,n}-\mu _i)|A_iA_i^T +\sigma _i^2 I_{d_g}|^{-\frac{1}{2}}(x_{i,n}-\mu _i)^T}\\&\Phi _{d_g+q}({\tilde{\Lambda }}_i^T |A_iA_i^T +\sigma _i^2 I_{d_g}|^{-\frac{1}{2}}(x_{i,n}-\mu _i) +{\tilde{\Lambda }}_i^T z_{i,n}|I_{d_g+q}-{\tilde{\Lambda }}_i^T{\tilde{\Lambda }}_i - \Lambda _i^T\Lambda _i). \end{aligned} \end{aligned}$$

Therefore, the conditional density function of $z_{i,n}$ given $x_{i,n}$ is as follows:

14 $$\begin{aligned} f(z_{i,n}|x_{i,n})= 2^{q} \phi _q(z_{i,n}) \frac{\Phi _{d_g+q}({\tilde{\Lambda }}_i^T |A_iA_i^T +\sigma _i^2 I_{d_g}|^{-\frac{1}{2}}(x_{i,n}-\mu _i) +{\tilde{\Lambda }}_i^T z_{i,n}|I_{d_g+q}-{\tilde{\Lambda }}_i^T{\tilde{\Lambda }}_i - \Lambda _i^T\Lambda _i)}{\Phi _{d_g}({\tilde{\Lambda }}_i^T |A_iA_i^T +\sigma _i^2 I_{d_g}|^{-\frac{1}{2}}(x_{i,n}-\mu _i) |I_{d_g}-{\tilde{\Lambda }}_i^T{\tilde{\Lambda }}_i)}. \end{aligned}$$

By combining Equations (13) and (14), we obtain the posterior logarithmic likelihood function of the complete data as follows:

15 $$\begin{aligned} \begin{aligned} \log {f(x_{i,n}, z_{i,n}|\theta _i)}&= c + \sum _{n=1}^{n_i}\{\sum _{g=1}^G [ \frac{d_g}{2} \log {|A_iA_i^T +\sigma _i^2 I_{d_g}|} + \frac{1}{2} tr((A_iA_i^T +\sigma _i^2 I_{d_g}))^{-1}\Vert x_{i,n}-\mu _i\Vert ^2 \\&\quad + \log [ \Phi _{d_g+q}({\tilde{\Lambda }}_i^T |A_iA_i^T +\sigma _i^2 I_{d_g}|^{-\frac{1}{2}}(x_{i,n}-\mu _i) \\&\quad +{\tilde{\Lambda }}_i^T z_{i,n}|I_{d_g+q}-{\tilde{\Lambda }}_i^T{\tilde{\Lambda }}_i - \Lambda _i^T\Lambda _i)] + \frac{1}{2} tr(\langle z_{i,n},z_{i,n}^T\rangle ) \}. \end{aligned} \end{aligned}$$

$$\square $$

## Methods

In this section, we present the detailed process and algorithm of parameter estimation and the DP algorithm with a privacy guarantee.

### Federated multi-views PPCA for non-Gaussian data

Based on the described analysis of the observed and latent variables, we use the EM and pseudo-Newton algorithms to estimate the parameters locally, release all the local parameters to the master, aggregate the parameters at the master, and estimate the global parameters using maximum likelihood estimation (Algorithm 1).
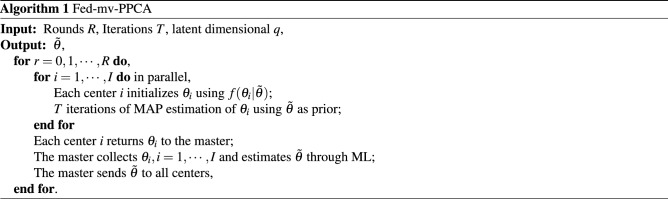


The parameter estimation in Algorithm 1 is as follows: For simplicity, we take the parameter $\mu$ as an example and assume that in center *i*, the $gth$-view is missing, instead of simply removing it. We assume $\forall i,g$.

16 $$\begin{aligned} \mu _i^{(g)} \sim N_{d_g}({\tilde{\mu }}^{(g)},{\sigma _{{\tilde{\mu }}}^2I_{d_g}}), \end{aligned}$$

**Step 1.** In each center: Estimate $\mu _i^{(g)}[s+1]|({\tilde{\mu }}^{(g)},{\sigma _{{\tilde{\mu }}}}^2)[s]$ from Equation (1). The marginal distribution of $x_{i,n}^{(g)}$ is as follows:

17 $$\begin{aligned} x_{i,n}^{(g)} \sim SN_{d_g}({\tilde{\mu }}_i^{(g)},\Sigma _i^{(g)},{\tilde{\Lambda }}_i^{(g)}), \end{aligned}$$

Recall:

18 $$\begin{aligned} f(x_{i,n}^{(g)} )=2(2\pi )^{-\frac{d_g}{2}}|\Sigma _i^{(g)}|^{-\frac{1}{2}}\exp {-\frac{1}{2}(x_{i,n}^{(g)}-\mu _i^{(g)})^T(\Sigma _i^{(g)})^{-1}(x_{i,n}^{(g)}-\mu _i^{(g)})}\Phi (({\tilde{\Lambda }}_i^{(g)})^T(\Sigma _i^{(g)})^{-1}(x_{i,n}^{(g)}-\mu _i^{(g)}) \end{aligned}$$

the log-likelihood function is:

19 $$\begin{aligned} {\mathfrak {L}}_i^{(g)}= & {} -\frac{1}{2}\{N_id_g\log {(2\pi )}\log {|\Sigma _i^{(g)}|}+\sum _{n=1}^{N_i}(x_{i,n}^{(g)}-\mu _i^{(g)})^T(\Sigma _i^{(g)})^{-1}(x_{i,n}^{(g)}-\mu _i^{(g)})+\nonumber \\{} & {} \zeta _0(({\tilde{\Lambda }}_i^{(g)})^T(\Sigma _i^{(g)})^{-1}(x_{i,n}^{(g)}-\mu _i^{(g)}))\}, \end{aligned}$$

where $\zeta _0(x)=\log {2x}$. Subsequently, we fixed the center order *i* and considered the following optimization problem for $\forall g\in {1,\cdots ,G}$,

20 $$\begin{aligned} \max _{\mu _i^{(g)}}{{\mathfrak {L}}_i^{(g)} } +\log {f(\mu _i^{(g)})}, \end{aligned}$$

where $\log {f(\mu _i^{(g)})} = -\frac{1}{2{\sigma _{{\tilde{\mu }}^{(g)}}}^2}(\mu _i^{(g)}-{\tilde{\mu }}^{(g)})^T(\mu _i^{(g)}-{\tilde{\mu }}^{(g)}) +c1,$ *c*1 is a constant independent of $\mu _i^{(g)}$. Using the maximum-likelihood method for this problem, we obtain the following:

21 $$\begin{aligned} \mu _i^{(g)}[s+1] = [N_iI_{d_g} + {\sigma _{{\tilde{\mu }}^{(g)}}}^{-2}[s]\Sigma _i^{(g)}]^{-1}[\sum _{i=1}^{N_i}x_{i,n}^{(g)} + {\sigma _{{\tilde{\mu }}^{(g)}}}^{-2}[s]\Sigma _i^{(g)}{\tilde{\mu }}^{(g)}[s] ]. \end{aligned}$$

**Step 2.** In the master: Estimate $({\tilde{\mu }}^{(g)}[s+1], {\sigma _{{\tilde{\mu }}^{(g)}}}^2[s+1])$ given $\mu ^{(g)}[s+1]$ for each *i*.

22 $$\begin{aligned} \begin{aligned} {\mathfrak {L}} =&\sum _{i=1}^I \log {f(\mu _i^{(g)})}\\ =&\sum _{i=1}^I\{c1 - \frac{1}{{\sigma _{{\tilde{\mu }}^{(g)}}}^2}\Vert \mu _i^{(g)}-{\tilde{\mu }}^{(g)}\Vert ^2 \}. \end{aligned} \end{aligned}$$

Let $\frac{\partial {\mathfrak {L}}}{\partial {\tilde{\mu }}^{(g)}} = 0,$ and $\frac{\partial {\mathfrak {L}}}{\partial {\sigma _{{\tilde{\mu }}^{(g)}}}^2} = 0$, we can obtain:

23 $$\begin{aligned} {\tilde{\mu }}^{(g)}[s+1] = I^{-1} \sum _{i=1}^{N_i} \mu _i^{(g)}[s+1], \end{aligned}$$

and

24 $$\begin{aligned} \sigma _{{\tilde{\mu }}^{(g)}}^{-2}[s+1] = (Id_g)^{-1}\sum _{i=1}^{N_i} \Vert \mu _i^{(g)}[s+1]-{\tilde{\mu }}^{(g)}[s+1]\Vert ^2, \end{aligned}$$

respectively.

We performed similar treatments for the other parameters. Notably, although the skewness parameter depends on both the location and scale parameters, the prior distribution of the transformed parameter $\delta$ is uniform under the Bayesian framework.

### DP-Fed-mv-PPCA algorithm for non-Gaussian data

In this section, we propose an improved **DP-Fed-mv-PPCA** algorithm based on clients with skewed normal data. Our main goal is to protect the privacy of the training parameters of the local data, which requires the use of different noise mechanisms for different components to perturb the data properly.

First, let us provide the basic concept of DP and classical combinatorial theory. Then, we will gradually use these theories in the algorithm.

#### Definition 1

Given a data universe ${\mathscr {X}}$, we say that two datasets $D,D'$ are neighbors if they differ by only one entry, which is denoted as $D {{\tilde{D}}}'\subseteq {\mathscr {X}}$. A randomized algorithm ${\mathscr {M}}$ is $(\varepsilon , \delta )$-differentially private (DP) if for all neighboring datasets $D,D'$ and for all events *S* in the output space of ${\mathscr {M}}$, we have ${\mathbb {P}}({\mathscr {M}}(D)\in S)\le e^{\varepsilon }{\mathbb {P}}({\mathscr {M}}(D')\in S) +\delta$.

#### Definition 2

Given a function $q: {\mathscr {X}}^n \rightarrow {\mathbb {R}}^d$, the Gaussian Mechanism is defined as: ${\mathscr {M}}(D;q;\cdot )=q(D) + Noise$; where *Noise* is drawn from a Gaussian distribution $N(0,\sigma ^2 I_d)$ with $\sigma \ge \frac{\sqrt{2\ln {1.25/\delta }}\Delta _2(q)}{\varepsilon }$, $\Delta _2(q)$ is the $l_2-$ sensitivity of the function *q*, i.e., $\Delta _2(q)=\sup _{D ~ D'}\Vert q(D)-q(D')\Vert _2$. The Gaussian mechanism preserves $(\varepsilon , \delta )-$DP.

#### Definition 3

Given a function $q: {\mathscr {X}}^n \rightarrow {\mathbb {R}}^d$, the Laplace mechanism is defined as: ${\mathscr {M}}(D;q;\cdot )=q(D) + Noise$; where *Noise* is drawn from a Laplace distribution $Lap(0,\frac{\Delta _1(q)}{\varepsilon }), \Delta _1(q)$ is the $l_1-$ sensitivity of the function *q*, i.e., $\Delta _1(q)=\sup _{D ~ D'}\Vert q(D)-q(D')\Vert _1$. The Laplace mechanism preserves $(\varepsilon , 0)-$DP.

#### Lemma 4.1

Given any function $q: {\mathscr {X}}^n \rightarrow {\mathbb {R}}^d$ and $\varepsilon >0, \delta \in (0,0.5)$, the Gaussian mechanism is defined as follows:

25 $$\begin{aligned} {\mathscr {M}}(D;q;\cdot )=q(D) + N({\varvec{0}},(\frac{(c+\sqrt{c^2 +\varepsilon })\Delta _2(q)}{\sqrt{2}\varepsilon })^2I_d), \end{aligned}$$

where $c=\sqrt{\ln ({2/(\sqrt{16\delta +1}-1)})}$ preserves $(\varepsilon , \delta )-$DP.

#### Lemma 4.2

Given any function $q: {\mathscr {X}}^n \rightarrow {\mathbb {R}}^{d\times q}$ and $\varepsilon >0, \delta \in (0,0.5)$, the matrix-normal mechanism is defined as follows:

26 $$\begin{aligned} {\mathscr {M}}(D;q;\cdot )=q(D) + N_{d,q}({\varvec{0}}_{d,q},(\frac{(c+\sqrt{c^2 +\varepsilon })\Delta _2(q)}{\sqrt{2}\varepsilon })^2I_d), \end{aligned}$$

where $c=\sqrt{\ln ({2/(\sqrt{16\delta +1}-1)})}$ preserves $(\varepsilon , \delta )-$DP.

#### Lemma 4.3

For $i=1,\cdots ,k$ let ${\mathscr {M}}_i$ be an $(\varepsilon _i, \delta _i)-$DP algorithm, and ${\mathscr {M}}(q)=({\mathscr {M}}_1(q),\cdots ,{\mathscr {M}}_k(q))$. Then, ${\mathscr {M}}$ is $(\sum _{i=1}^k\varepsilon _i, \sum _{i=1}^k\delta _i)-$DP.

Although the algorithm does not show an expression for client-level optimization, we can ensure that the local parameters achieve DP with appropriate clipping methods, such as Zhang et al.^31^ (see Algorithm 2 for the detailed procedure).
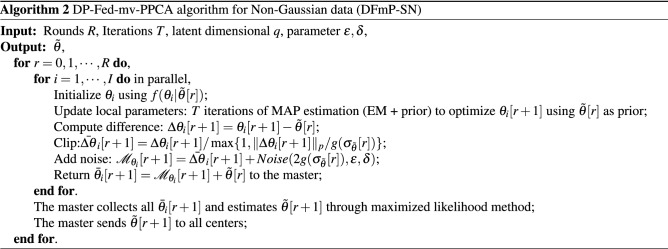


With respect to Algorithm 1, we need to perform appropriate clipping when the local dataset uses the EM and textbfpseudo-Newtonalgorithms to estimate the parameters, and then add noise from different mechanisms (Laplacian or Gaussian^32^) to the parameters to ensure that the data released to the master achieve DP^33^. The specific execution process is as follows:

1. The client provides the prior distribution in round r and updates the local parameters according to Algorithm 1 based on the prior distribution. The client calculates the difference between the updated parameters in round r + 1 and the prior parameters in round r.

27 $$\begin{aligned} \Delta \theta _i[r+1]= \theta _i[r+1]-{\tilde{\theta }}[r]. \end{aligned}$$

2. The updated difference is clipped according to the standard deviation of the prior values.

28 $$\begin{aligned} \bar{\Delta \theta }_i[r+1] = \Delta \theta _i[r+1]/\max \{1,\Vert \Delta \theta _i[r+1]\Vert _p/g(\sigma _{{\tilde{\theta }}}[r])\}, \end{aligned}$$

where $g(\sigma _{{\tilde{\theta }}}[r]):= c2\cdot {\tilde{\theta }}[r]$ and *c*2 is a constant fixed by the client. The clipping upper bound of $\ell _p$ norm of $\bar{\Delta \theta }_i[r+1]$ is $g(\sigma _{{\tilde{\theta }}}[r])$, and the $\ell _p$ sensitivity of $\bar{\Delta \theta }_i[r+1]$ at most $2g(\sigma _{{\tilde{\theta }}}[r])$.

3. We propose a noise mechanism for the clipped difference using

29 $$\begin{aligned} {\mathscr {M}}_{\theta _i}[r+1]:=\bar{\Delta \theta }_i[r+1] + Noise(2g(\sigma _{{\tilde{\theta }}}[r]),\varepsilon ,\delta ). \end{aligned}$$

We propose to add Gaussian noise to $\bar{\Delta \mu _i}^{(g)}$ and $\bar{\Delta A_i}^{(g)}$, while adding Laplace noise to $(\bar{\Delta \sigma }^{(g)})^2_i$ and ${\bar{\Delta }}\delta (\lambda )_i^{(g)}$.

4. The client adds a priori and sends $\bar{\Delta \theta }_i[r+1] = {\mathscr {M}}_{\theta _i}[r+1] + {\tilde{\theta }}[r]$ to the master.

**Figure 1:**
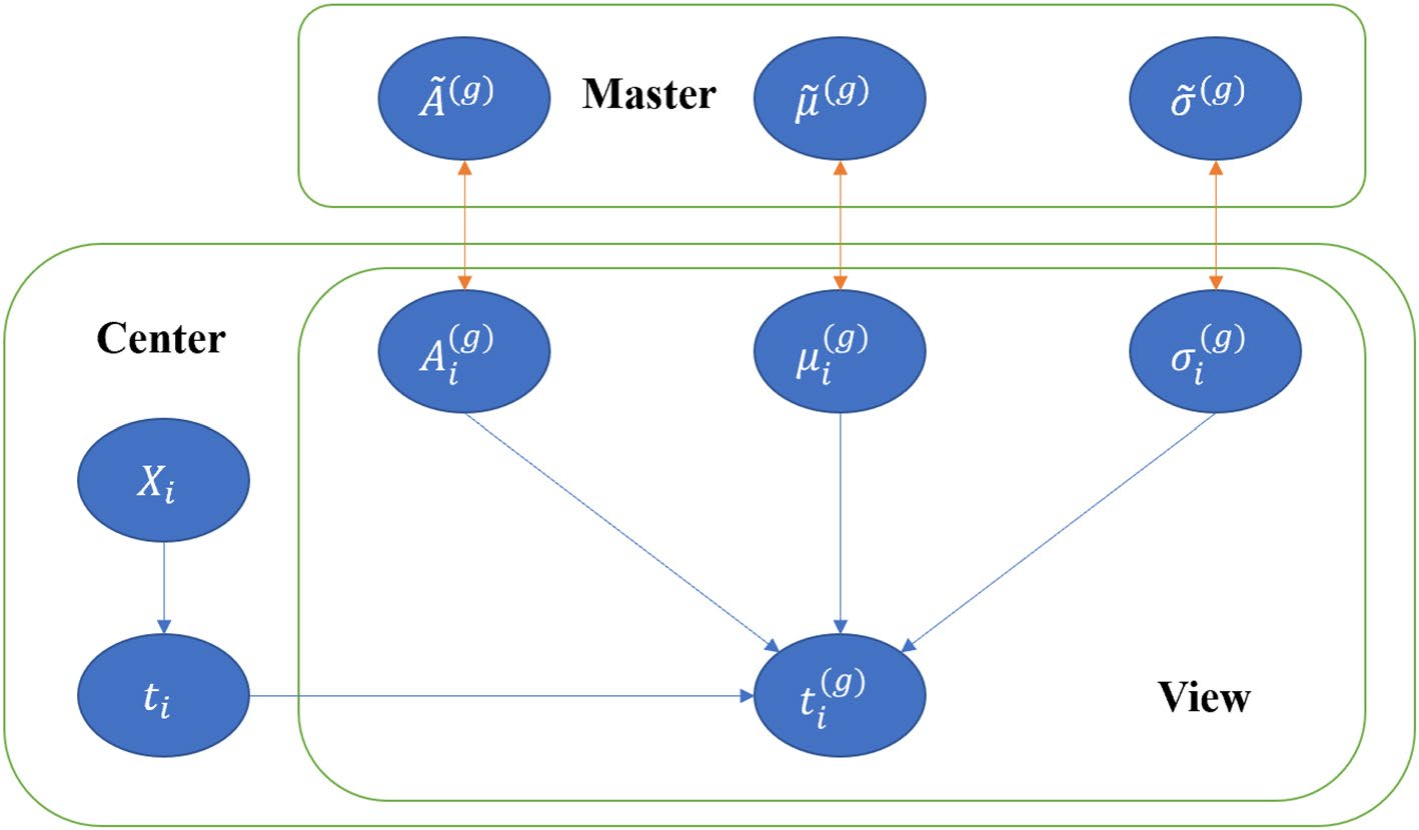
Graphical model of Fed-mv-PPCA.

**Figure 2:**
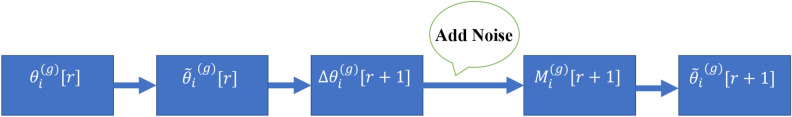
Parameter iteration process of algorithm DFmP-SN.

Figures 1 and 2 shows the graphical model of Fed-mv-PPCA and iterative flow diagram of component parameters of the skew normal mixture model.

We provide the privacy budget for Algorithm 1 using the following theorem:

#### Theorem 4.4

For simplicity, let us select the same $\varepsilon ,\delta$ for all the mechanisms considered (a generalization to a parameter-specific choice of $\varepsilon _i,\delta _i$ is straightforward). The total privacy budget for the output of Algorithm 2 is $(4G\varepsilon ,3G\delta )$, where *K* denotes the total number of views.

Notably, the data in each center were disjoint. At all centers, we consider the mechanism ${\mathscr {M}}:= \{ {\mathscr {M}}_{\mu _i^{(g)}}, {\mathscr {M}}_{A_i^{(g)}}, {\mathscr {M}}_{{\sigma _i^{(g)}}^2},{\mathscr {M}}_{\delta (\lambda )_i^{(g)}} \}$, where for all *g*, ${\mathscr {M}}_{\mu _i^{(g)}}$ and ${\mathscr {M}}_{A_i^{(g)}}$ are $(\varepsilon ,\delta )$-DP, whereas for all *g*, ${\mathscr {M}}_{{\sigma _i^{(g)}}^2}$ and ${\mathscr {M}}_{\delta (\lambda )_i^{(g)}}$ are $\varepsilon$-DP. We can obtain the results from the composition theorem and post processing of DP.

## Performance evaluation

### Introduction to dataset

Synthesis data (SD): A total of 10,000 observations were generated using the generation model, which comprised $$k=3 $$ views with dimensions of $$d_1=2, d_2=10$$, and $$d_3=5$$. Each view was generated using a common 5-dimensional potential space. We selected the parameters $$A^{(g)},\mu ^{(g)},\sigma ^{(g)}$$ randomly.

Didi Chuxing GAIA data (DiDi): The data security of connected car users is a concern. In this section, as in [9], we use car-networking data from Didi Chuxing GAIA Initiative (https:gaia.didichuxing.com). We selected 10,000 vehicle data points from the driver trajectory data of the Chengdu Second Ring Road from 00 : 00 to 24 : 00 on October 15, 2019. The longitude and latitude coordinates in the dataset were also converted through WGS84 coordinate system and thereafter converted into east-north coordinates in meters using the UTM grid point system. We also used the binary Gaussian random error in [9].

### Results

To verify that the non-Gaussian data fitting is more effective in the actual data, in the DiDi dataset, we first performed a mixed biased normal distribution fitting on the data and compared the fitting effect when the number of components was 1-8, which significantly improved the accuracy of the normal fitting compared with [9]. From the results, the best fit was achieved when the component was $g=5$. For the comparison index of the experiment, we used the common evaluation indices MSE and mean absolute error (MAE). Further results are summarized in Table 1 along with AIC and BIC results, which are caused by the complex traffic condition data structures.

To verify that our algorithm can provide both privacy assurance and data availability, we compared our method with Fed-mv-PPCA in the SD and DiDi datasets and with the FedVCP method in the dataset.

**Figure 3:**
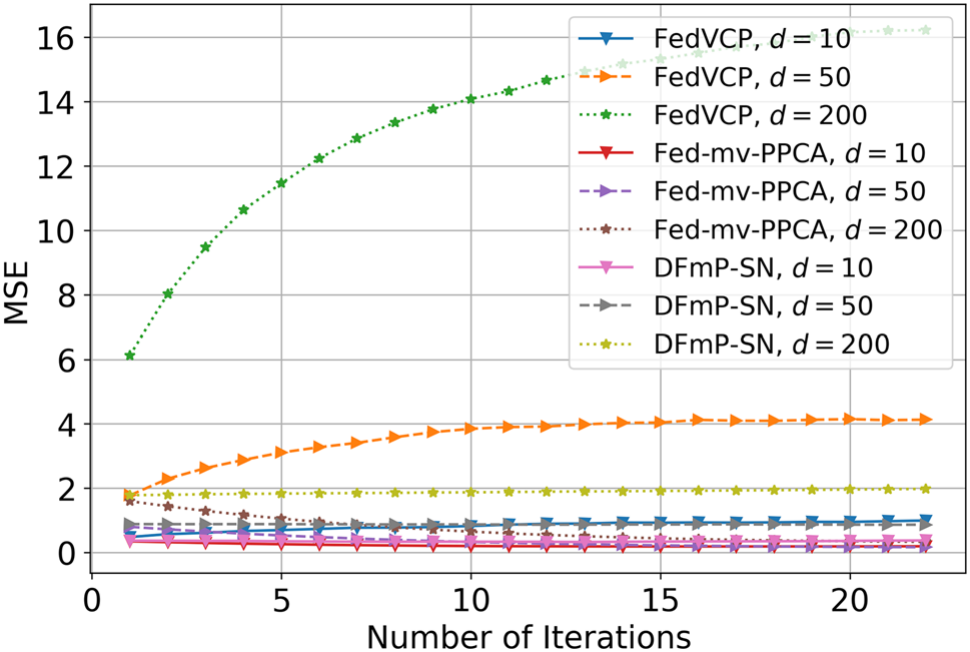
Effect comparison of three algorithms in different dimensions of MSE (FedVCP, Fed-mv-PPCA, DFmP-Sn) on DiDi dataset, $\varepsilon =0.1$.

**Figure 4:**
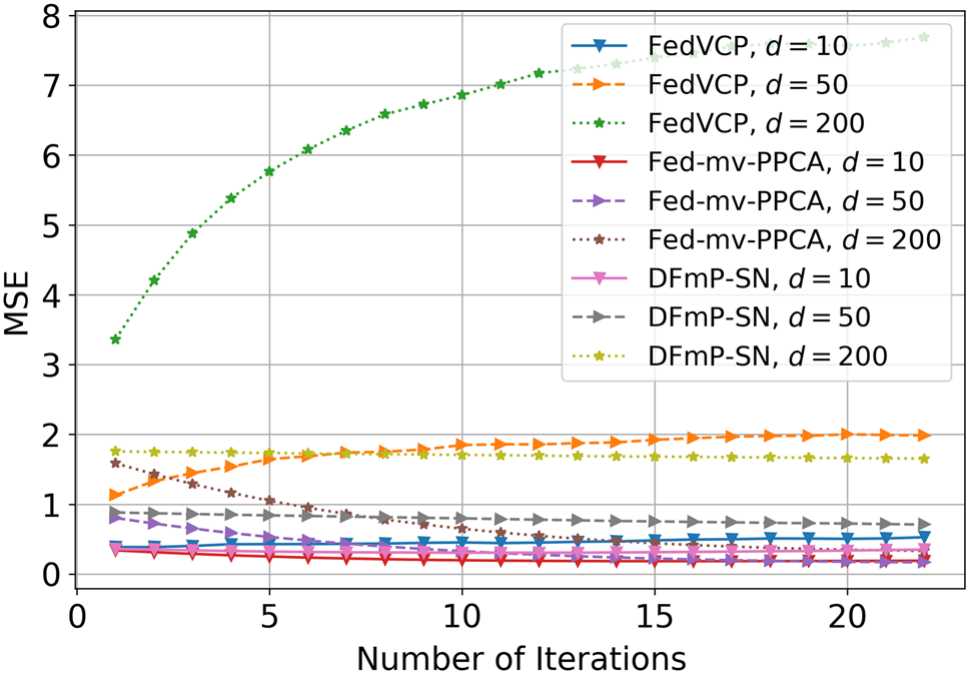
Effect comparison of three algorithms in different dimensions of MSE (FedVCP, Fed-mv-PPCA, DFmP-Sn) on DiDi dataset, $\varepsilon =0.2$.

**Figure 5:**
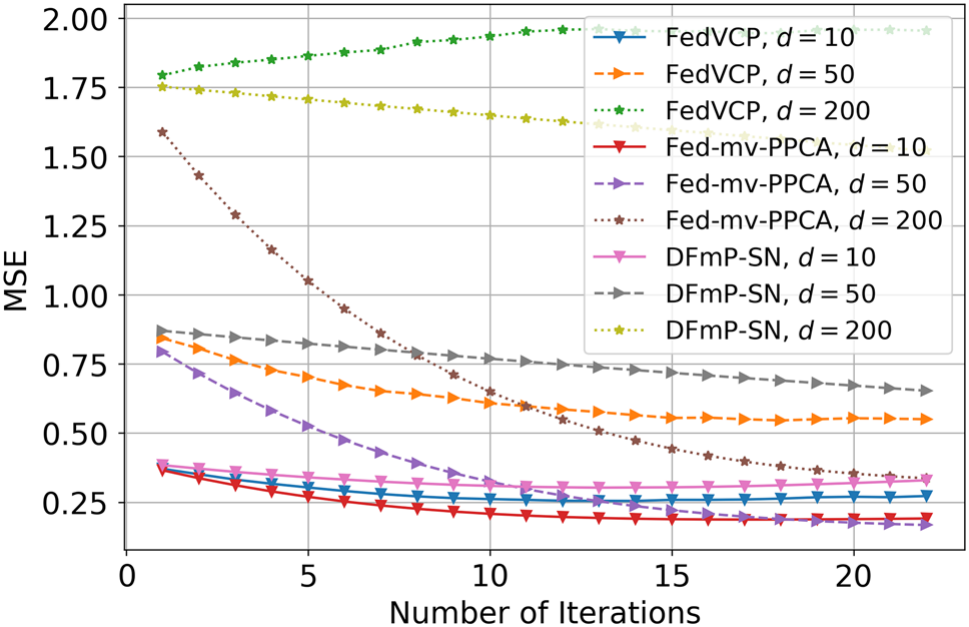
Effect comparison of three algorithms in different dimensions of MSE (FedVCP, Fed-mv-PPCA, DFmP-Sn) on DiDi dataset, $\varepsilon =0.8$.

**Figure 6:**
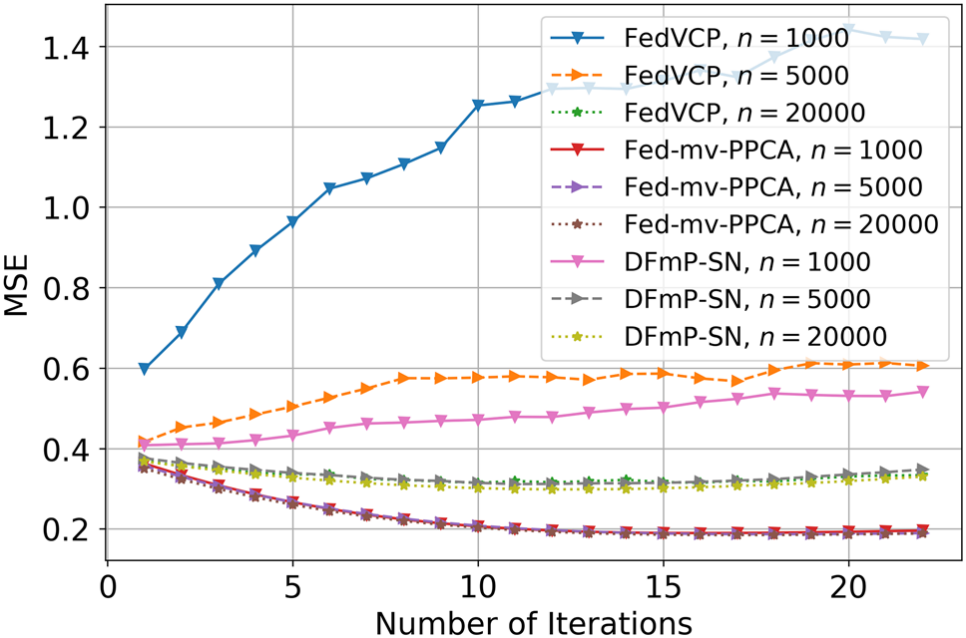
Effect comparison of three algorithms in different samples of MSE (FedVCP, Fed-mv-PPCA, DFmP-Sn) on DiDi dataset, $\varepsilon =0.1$.

**Figure 7:**
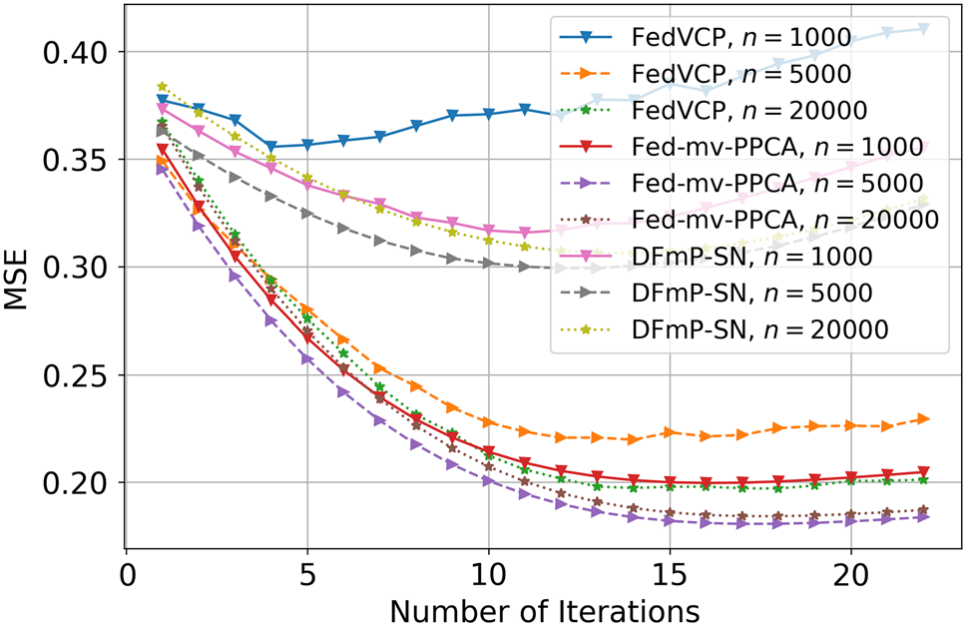
Effect comparison of three algorithms in different samples of MSE (FedVCP, Fed-mv-PPCA, DFmP-Sn) on DiDi dataset, $\varepsilon =0.2$.

**Figure 8:**
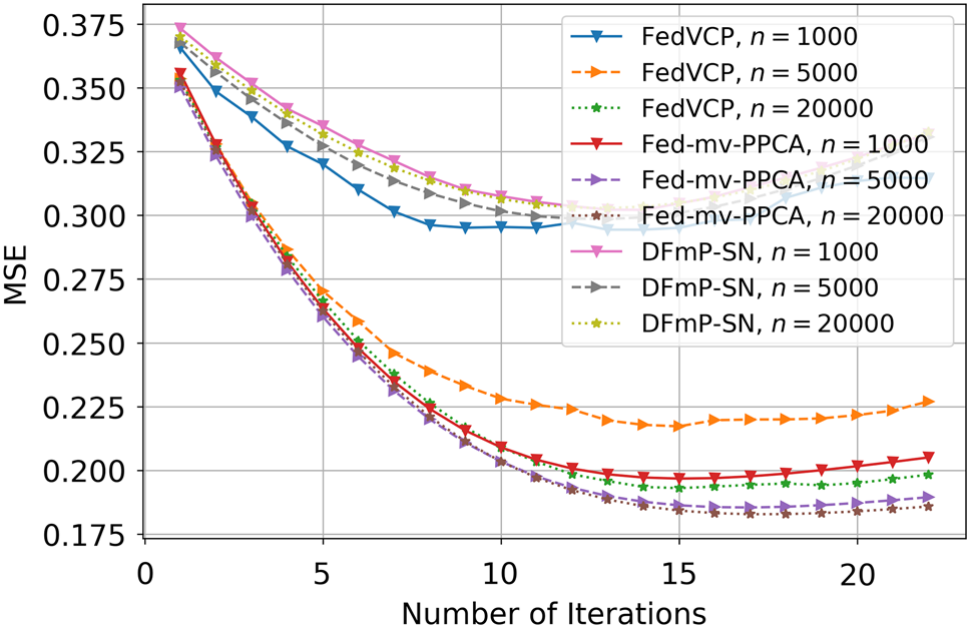
Effect comparison of three algorithms in different samples of MSE (FedVCP, Fed-mv-PPCA, DFmP-Sn) on DiDi dataset, $\varepsilon =0.8$.

**Figure 9:**
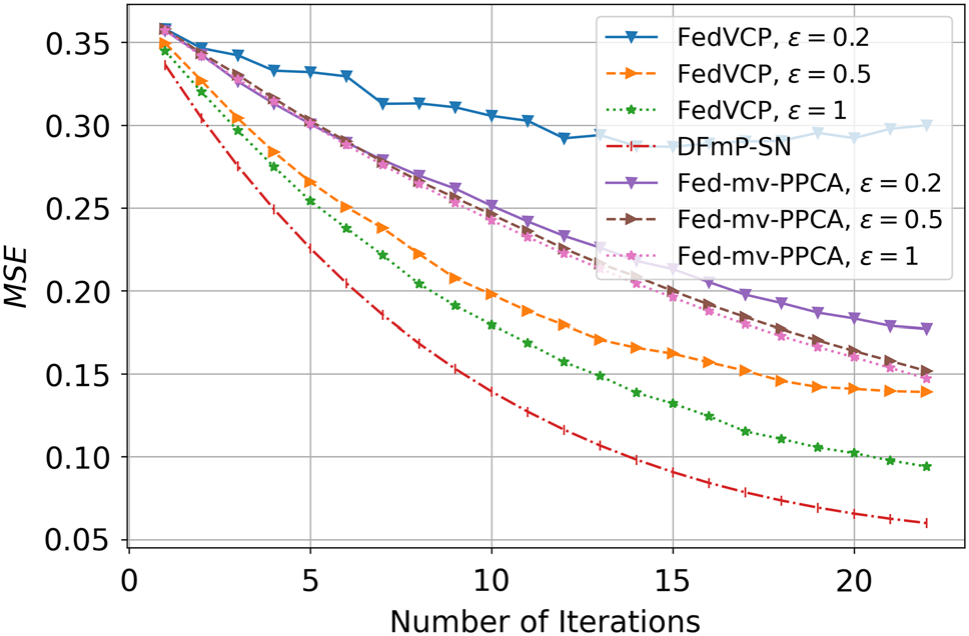
Effect comparison of three algorithms in different budget of MSE (FedVCP, Fed-mv-PPCA, DFmP-Sn) on DiDi dataset, $d=10$.

For the SD dataset and DiDi dataset, the calculation results (see Tables 1 and 2) demonstrate that for different privacy parameters, our algorithm can achieve privacy guarantee and is clearly better than FedVCP. For the DiDi dataset, our algorithm achieved a stronger privacy guarantee. It can be clearly seen from Figs. 3, 4, 5, 6, 7, 8 and 9 that our estimation error is smaller in MSE .

**Table 1:** Results of model selection for gaussian and non-gaussian on SD dataset.

	1	2	3	4	5	6	7	8
**k (Gaussian)**								
AIC	405.12	415.11	427.55	433.84	439.07	434.91	430.10	431.78
BIC	432.22	445.32	451.29	470.31	481.19	467.28	472.23	473.18
MSE	0.21	0.19	0.15	0.14	0.13	0.12	0.12	0.12
MAE	0.19	0.14	0.13	0.11	0.07	0.07	0.07	0.06
**k (Non-Gaussian)**								
AIC	472.11	477.36	479.35	480.85	490.01	487.75	476.68	477.79
BIC	500.15	521.12	522.23	531.11	543.33	540.59	540.01	522.55
MSE	0.09	0.07	0.07	0.04	0.02	0.02	0.02	0.02
MAE	0.07	0.06	0.06	0.03	0.01	0.01	0.01	0.01

**Table 2:** Results of model selection for gaussian and non-gaussian on DiDi dataset.

	1	2	3	4	5	6	7	8
**k (Gaussian)**								
AIC	1789.25	1792.11	1840.22	1823.33	1811.05	1766.55	1744.56	1705.63
BIC	1822.05	1827.66	1859.87	1845.55	1833.78	1821.25	1801.01	1796.34
MSE	0.17	0.11	0.09	0.09	0.09	0.09	0.09	0.07
MAE	0.19	0.12	0.10	0.10	0.10	0.10	0.09	0.09
**k (Non-Gaussian)**								
AIC	1925.23	1935.53	1979.99	1976.52	1927.27	1900.35	1892.25	1888.87
BIC	1975.57	1986.68	2016.64	2008.98	2001.13	1999.76	1989.68	1980.35
MSE	0.11	0.10	0.04	0.04	0.03	0.03	0.02	0.02
MAE	0.05	0.04	0.02	0.02	0.012	0.01	0.01	0.01

## Conclusions

In this study, we introduce a skewed normal distribution as the non-Gaussian data likelihood and prior. Our application proves that **Fed-mv-PPCA** is robust to increasing levels of heterogeneity in training centers, and provides high-quality data reconstruction that outperforms competing methods in all scenarios. In addition, when DP was introduced, we investigated the performance of the proposed method according to different privacy budget scenarios. Notably, there are four DP hyperparameters that play a key role and may influence the properties of **DP-Fed-mv-PPCA**: the privacy budget parameters $(\varepsilon ,\delta )$, and the clipping and skewness parameters $\delta (\lambda )$. These parameters are closely related and both help determine the magnitude of the noise used to perturb the updated difference $\Delta \theta$. Indeed, increasing $\delta$ or decreasing the multiplicative constant in the clipping mechanism implies the addition of less noise, thereby improving the overall utility of the global model. However, the smaller the values of $\varepsilon$ and $\delta$, the higher the privacy guarantee.

There is much more work to be conducted in the future. First, reducing the computational and sample complexities of the DP calculation between the client and server is the key problem because heterogeneous data with multiple sources often face the problem of small data. Second, different noise mechanisms are also the main direction of subsequent studies, and we note that matrix variables and tensor data are also challenges in modern FL. Finally, we studied distributions with thicker tails and shear methods suitable for high-dimensional data to improve the robustness of the model. Meanwhile, for outlier datasets, the Huber contamination model can be considered for the FL of DP.

## Data Availability

The datasets generated and/or analyzed in the present study are not publicly available, but are available from the corresponding author upon reasonable request.
